# Sialolithiasis—Do Early Diagnosis and Removal Minimize Post-Operative Morbidity?

**DOI:** 10.3390/medicina56070332

**Published:** 2020-07-02

**Authors:** Gal Avishai, Yehonatan Ben-Zvi, Omar Ghanaiem, Gavriel Chaushu, Hanna Gilat

**Affiliations:** 1 Department of Oral and Maxillofacial Surgery, Rabin Medical Center, 49414 Petach-Tikva, Israel; yonident@gmail.com (Y.B.-Z.); gabi.chaushu@gmail.com (G.C.); 2 The Maurice and Gabriela Goldschleger School of Dental Medicine, Tel-Aviv University, 69978 Tel Aviv, Israel; omarghanaiem@gmail.com; 3 Department of Otolaryngology-Head and Neck Surgery, Rabin Medical Center, 69978 Petach Tikva, Israel; Hannagi2@clalit.org.il

**Keywords:** sialolithiasis, sialendoscopy, sialolith, morbidity

## Abstract

*Background and objectives:* Sialolithiasis is an inflammation of a salivary gland due to obstruction of salivary flow by a sialolith. We aim to assess potential factors that may predict lower morbidity following endoscopically assisted per-oral sialolith removal. *Materials and Methods:* Retrospective cohort study. Retrospective review of 100 records of patients with sialolithiasis, following surgical sialolith removal. A single medical center (Department of oral and maxillofacial surgery-Rabin Medical Center, Beilinson & Hasharon–Israel) survey. Data were gleaned from the patient files based on a structured questionnaire. Factors that may predict morbidity were evaluated using linear regression equation. *Results:* 59 of the subjects were men and 41 were women. The mean age of the patients in the study was 50 ± 17.5 years. Sialolith volume and past antibiotic treatment were positively associated while age was negatively associated with hospitalization duration. *Conclusion:* Early sialolith diagnosis and removal may lower postoperative morbidity.

## 1. Introduction

Sialoliths are calcified structures located in the ductal system or the parenchyma of the salivary gland. Sialoliths vary in size and weight (range 1 mg to 6 grams; average 300 mg) [1]. In general, submandibular sialoliths are bigger than parotid ones [2]. Approximately 59% of all sialoliths have a diameter between 2.1–10 mm and 7.6% are larger than 15 mm in section [3]. Giant sialoliths (> 2 cm) are most commonly found in the submandibular gland body rather than in the major ducts [4].

The specific pathogenesis of sialoliths is not completely understood. Two major contributing factors were proposed: 1. the anatomical structure of the gland and its ducts; 2. saliva’s chemical composition [5]. A popular theory claims that sialoliths start as microscopic (Sialomicrolith) calcification of crystals containing calcium, phosphorus and other organic materials in the salivary glands. The incidence of sialomicroliths is associated with age and secretory inactivity. These microstructures might fuse together to form a sialolith [6,7].

Sialolithiasis (inflammation of a salivary gland due to a sialolith) is a common disorder that affects the salivary glands and may be characterized by the obstruction of the salivary secretion [8]. Most individuals presenting with a sialolith will have symptoms of sialolithiasis. Sialolithiasis symptoms include: pain, ill-tasting secretion and swelling of the obstructed gland, which usually appear before or during mealtime. When the gland is obstructed it is susceptible retrograde bacterial infection from the oral flora, which may lead to systemic infection and pus secretion from the salivary duct. These symptoms are characterized by their episodic appearance and a patient with a sialolith may have periods of painful symptoms followed by variable periods of no symptoms [9]. CT imaging is recommended to determine exact size and location of the sialolith, and will aid in surgery.

State-of-the-art treatment of sialolithiasis focuses on organ preservation. Minimally invasive intervention is recommended as the first line of treatment. Sialoliths that are large (> 3 mm) and located proximal to the gland may be retrieved endoscopically or surgically by an intraoral approach [10]. Sialendoscopy may use an intraductal camera and a basket retriever, intracorporal shockwave lithotripsy. If minimally invasive methods fail, the entire gland needs to be removed surgically [11]. Surgical treatment may be intraoral or extraoral, may include the extraction of the sialolith or excision of the gland itself [12].

The purpose of the present patient record analysis was to assess potential factors that may minimize morbidity following endoscopically assisted per-oral sialolith removal.

## 2. Materials and Methods

Retrospective cohort analysis at a single medical center—Department of Oral and Maxillofacial Surgery—Rabin Medical Center, Belinson & Hasharon-Israel.

### 2.1. Inclusion Criteria

Patients treated for removal of sialoliths between 2013 and 2019; existing preoperative imaging allowing assessment of sialolith size and location; sialolith location—in the gland itself or proximal to the middle portion of the excretory system; sialolith size—diameter > excretory duct diameter; minimally invasive intraoral surgical approach; general anesthesia. 

### 2.2. Exclusion Criteria

Insufficient data; local anesthesia; mobile sialolith; sialolith size—diameter smaller than 3 mm. 

### 2.3. Data Collection

**Figure 1:**
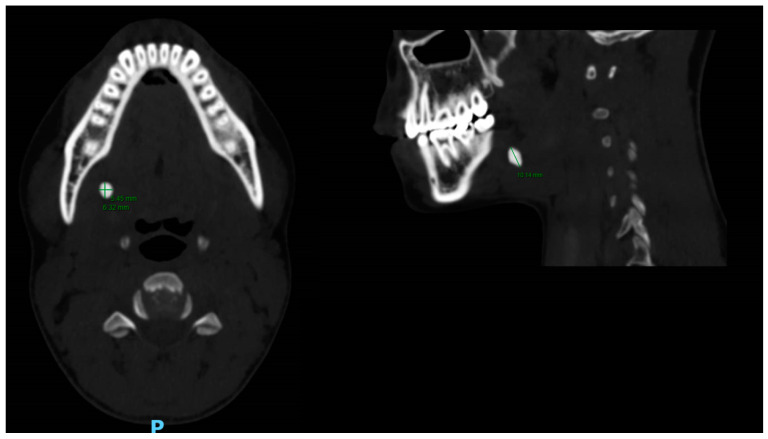
Computed Tomography (CT) of a left submandibular gland sialolith, sagittal section (right), 10.14 mm of the maximal superior-inferior dimension, transverse section (left), dimensions: 5.45 mm latero-medial by 6.32 mm anterior-posterior.

**Figure 2:**
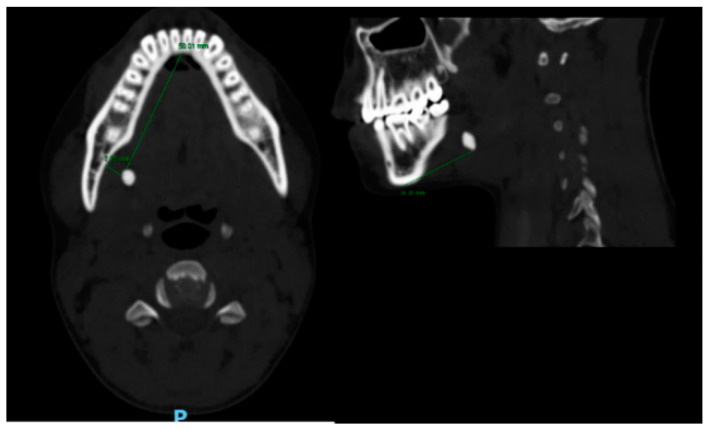
Computed Tomography (CT) of a right submandibular gland sialolith, The stone’s distance from the lingual border of the mandible (7.25 mm) and the anterior midline of the mandible (50.01 mm) (left), The stone’s distance from the inferior border of the mandible (34.26 mm) (right).

**Figure 3:**
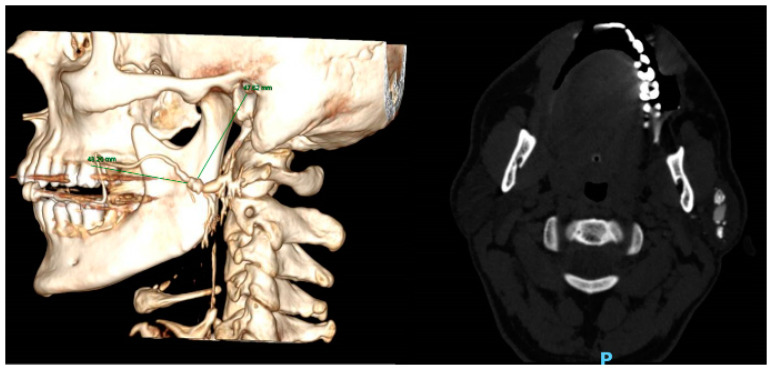
Computed Tomography (CT) Sialography of a left protid gland sialolith. Showing distance of stone from external acoustic meatus (47.62 mm) and from the second molar (43.20 mm).

(a) Data was collected using a structured questionnaire from the patient’s files for all patients who fulfilled the inclusion criteria in a consecutive manner:Demographic features: gender; age; medical history; medications; allergies.
(b) Primary complaint
(c) Symptoms: mealtime related swelling; purulent secretion; salty tasting saliva; number of episodes; previous antibiotic treatment prescribed.
(d) Physical examination: swelling; sensitivity; pain; purulence; palpability.
(e) Imaging modality used
(f) Sialolith characteristics (according to CT)
(g) Size (Figure 1): length; width; height; volume (calculated by: length X width X height, we used the formula for a volume of a box as an approximation for the volume of the irregularly shaped sialoliths).
(h) Anatomical location, distance from sialolith to anatomical landmark (Figure 2 and Figure 3)
(i) Operation: duration; outcome; hospitalization days; need for second attempt surgery.

### 2.4. Statistical Analysis

Descriptive analysis of all parameters included mean and standard deviations for all quantitative variables. 

In order to predict morbidity (duration of patient’s hospitalization) a linear regression equation was used, based on the information collected on dependent variables for each patient before and during treatment. In order to examine normal distribution of dependent variable “Hospitalization days” we used Shapiro-Wilk’s test.

## 3. Results

All the examined parameters are summarized in Table 1. 

### 3.1. Demographic Features

One hundred patients (59 males and 41 females; Age range 19–85 years, mean age 50 ± 17.5) were included. American Society of Anesthesiologists (ASA) physical status classification was used for assessment of the medical status; I-62%, II-33%, III-5%. Medications were used by 38%. Only 17 patients (17%) suffered from one or more drug allergy. 

### 3.2. Primary Complaint

Thirty percent, of the patients suffered from mealtime related swelling in the past, 9% suffered from purulent saliva, 12% complained about pain sensation and only 2% experienced salty tasting saliva. Swelling episodes ranged from; 23% once, 23% twice, 29% more than three swelling episodes and 25% of the patients never had a swelling episode in the past. Seventeen percent needed an antibiotic treatment in the past.

### 3.3. Physical Examination 

On the day of administration 49% of the patients suffered from swelling, 32% experienced pain on palpation. Fifty percent of the glands secreted normal and pure saliva while 2% with had plaque, more than 12% purulent saliva and 36% with no saliva secretion. Sixty-five percent of the sialoliths were palpable.

### 3.4. Imaging Modality Used

Most of the sialoliths (48%) were diagnosed using ultra sound (US) and 7% by panoramic x-ray. In 87% computed tomography (CT) scans were used. More than half (51%) of the patients required two or more imaging modalities to complete the diagnosis and treatment.

### 3.5. Gland Involved

The submandibular gland was involved in 94 patients (94%) and the parotid gland in 6 patients (6%). Cases of the sublingual and minor salivary glands were not included. Distribution between the right (48%) and left sides (52%) was almost equal, with no bilateral involvement. 

### 3.6. Sialolith Characteristics (according to CT)

The average dimensions of all sialoliths were; 6.6 mm in anterior- posterior dimension, 7.4 mm in superior-inferior dimension and 5.2 mm in lateral-lateral dimension. The mean diameter was 7.7 mm. Regarding anatomical location for both the parotid and submandibular glands: 12% were located in the gland itself, 44% in the hillus, 27% in the main duct and 17% toward the papilla. Mean landmark location for submandibular sialoliths was: 7.9 mm from the lingual mandibular border; 31 mm from the inferior border and 39 mm from the anterior (symphysis). Parotid sialoliths were located 41.8 mm from the CEJ (cemento-enamel junction) of the second maxillary molar and 39.6 mm from the external acoustic meatus.

### 3.7. Operation

All patients went through an attempt to remove the sialolith surgically (mean duration 50 ± 15.6 min) under general anesthesia using an intraoral approach.

### 3.8. Success or Failure in Sialolith Removal 

Of the 100 cases participated in the study, we were able to trace the final state of 95. Twelve percent of surgeries ended with partial success or complete failure in stone removal and 87% of surgeries ended with the removal of the whole stone. Eleven cases required a second attempt.

### 3.9. Morbidity Expressed by Hospitalization Days

Data about hospitalization days was available for 90 sialoliths. The mean hospitalization duration after surgery was 2.57 ± 0.82 days, ranging from 1 to 5 days. In order to examine whether the dependent variable “Hospitalization days” is normally distributed, we used Shapiro-Wilk’s test. The results showed no significance (p.v = 0.149) and therefore we cannot reject the Null Hypothesis which claims the data is normally distributed. Postoperative complications were found in 5 of the 90 patients reviewed (5.5%) including infection (4 patients, 4.45%) and lingual nerve neurological deficit (1 patient, 0.011%). These complications were not found to have a significant statistical correlation to the number of hospitalization days. A linear regression was used to predict the number of hospitalization days, based on the information collected on dependent variables for each patient before and during treatment. The variables included: age; gender; medications; drug allergy; complaints; number of past swelling episodes; past antibiotic consumption; gland swelling; pain; purulence; palpation; sialolith anatomic location; imaging; number of imaging studies; sialolith volume; gland side; surgery duration; postoperative complications, surgical outcome.

When all variables were included in the linear regression analysis, they were able to account for 82.5% of the variance of the hospitalization days, but the whole model was not found to be significant F (19, 10) = 2.485, *p* > 0.05). (Table 2a.)

After removing variables with high correlation between predictors and variables (VIF [Variance Inflation Factor] greater than 5), that significantly impaired the model, five variables in the final model were found to be significant and together they form a significant model for predicting the number of hospitalization days. The variables included in the final model were surgery duration; sialolith volume; past antibiotic treatment; gland side; age. These five variables accounted for 59% of the variance of hospitalization days and maintained a statistically significant model (F (5, 26) = 7.477, *p* < 0.001). (Table 2b.)

The model found that the variables: surgery duration, sialolith volume and past antibiotic treatment were positively associated with duration of hospitalization. Longer surgery, increased sialolith volume and more antibiotics consumption in the past will increase the number of hospitalization days. Variables age and sialolith location on the left side are negatively associated with the number of hospitalization days.

## 4. Discussion 

In the present study, 100 cases of endoscopically assisted per oral sialolith removal were reviewed. Available information included demographics, disease manifestation and treatment. 

### 4.1. Demographic Features

There was no significant difference in distribution between genders. Some studies report similar equal distribution [3] while others described a male predominance [13].

Escudier et al. [2] described a high incidence of sialolithiasis at ages 25 to 50 years. Lustmann et al. [3] found that sialoliths are most prevalent during 3rd to 6th decades of life. Age of onset in (50 ± 17.5) is confirmed in the present study. Pediatric sialolithiasis represents 3% of all sialolithiasis cases [3]. In the present study all patients are adults (19–85 years).

### 4.2. Primary Complaint and Symptoms

Swelling is the most frequent symptom found, pain is the second-most [2,3,4]. The results of the present study are consistent with these clinical findings. Some sialoliths were discovered incidentally, this is likely due to the slow nature of sialolith growth and to the fact that symptoms arise when the drainage of the gland is blocked [9]. It is not surprising that in the present study 65% of the sialoliths were palpable and more than 50% of the glands secreted normal clear saliva on examination.

### 4.3. Imaging Modality

Ugga et al. [14] describe that US represents an excellent first choice diagnostic technique. In addition, CT, MRI and MR sialography can be reserved to patients with negative or inconclusive US results and a clinical presentation suggesting ductal obstruction. In the present study, the main imaging modalities used were US and CT. More than half (51%) of the patients required two or more imaging modalities to complete diagnosis and treatment.

### 4.4. Gland Involved and Sialolith Characteristics

In the present study, as reported in the literature [5], the submandibular was by far more involved compared to the parotid gland. Most (44%) sialoliths were found to be in the hilus of the gland or in the proximal duct system (27%). The minority of the sialoliths (17%) were within the distal duct system and 12% were located intraparenchymal. Previous studies also described predominant sialolith distribution in the glands in the hilum and the proximal duct system [15].

Sialolith diameter varied; (38.8%) smaller than 5 mm, (44.7%) 5–8 mm and (16.4%) bigger than 8 mm in diameter. Lustmann et al. reported a diameter of 5–10 mm [3].

Submandibular gland sialolith location in the duct was described by Foletti et al. [16]. The submandibular gland excretion system is categorized into unequally long sections: distal third–anterior to the lingual nerve; a proximal third which is within the gland; and a middle third, between them. Intraoral approach is recommended for anterior sialoliths and middle third sialoliths.

Equal distribution between right and left was also observed and previously reported [17]. This finding can be attributed to the high level of symmetry in the parameters of bilateral submandibular glands [18]_._

### 4.5. Treatment Approach and Hospitalization

The differing size of sialoliths, as well as their location in the gland`s duct systems, is surgically significant. As published by Zenk et al. [19] some of the sialoliths could only be removed using an endoscopic procedure. In addition, they mentioned that 92% of the submandibular gland sialoliths may be treated by intraoral approach. In the present study, all submandibular gland sialoliths were treated by intraoral approach under general anesthesia.

A significant positive relationship between hospitalization days to young age, surgery duration, sialolith size and antibiotic treatment prior the surgery was established. These variables infer that diagnosis and treatment at a later age, when sialoliths are bigger and patients have already experienced infection, yields a more complex case, requiring more complicated surgery. Consequently, increased postoperative morbidity (longer hospitalization) will be experienced. Shigeishi et al. [20] reported that prolonged operation duration was associated with postoperative complications after oral surgery and prolonged hospitalization. Surgery duration affected surgical infection rate and blood loss in patients following head and neck surgery [21]. On the contrary, others [22] found no significant relationship between operation time and hospitalization days in head and neck patients. Even though literature remains controversial whether operation time is a risk factor for complications following oral surgery and lengthens hospitalization, the present study emphasizes the importance to reduce operation time by early diagnosis and treatment.

There are many other factors that may affect the recovery period of the patients, recent studies by Isola et al. have demonstrated the importance of nutraceutical agents on the healing processes and inflammatory reactions in the oral cavity [23,24].

The present data shows that patients who needed antibiotics prior to surgery would probably need to stay in the hospital longer after the operation. There is some controversy about the timing of sialolith removal in cases presenting with acute inflammation. Some clinicians [25] insist that immediate surgical removal is contraindicated, since there is risk of spreading the infection, making the surgery unnecessarily difficult and extend hospitalization. Others [3,4], claimed that surgery should not be delayed and that sialolith should be removed as soon as possible because most symptoms result from salivary flow obstruction. The present study suggests that early removal might improve outcome.

The present study demonstrated a significant negative relationship between hospitalization days and the side of sialolith location. Both operating surgeons were right-handed and surgical access on the left side was easier for them. A literature search of right vs. left sided operations did not yield articles reporting similar discrepancy.

## 5. Conclusions

In endoscopically assisted per-oral sialolith removal, surgery duration, sialolith volume and past antibiotic treatment correlate with increased duration of post-operative hospitalization time.

## Figures and Tables

**Table 1:** Summary of Examined Parameters.

Parameter	Value
Demographics	
Male: female (ratio)	59:41
Age (mean (years), SD)	50 ± 17.5
Age range (years)	19–85
ASA I, II, III (%)	62, 33, 5
Drug allergies (%)	17
Main complain	
Mealtime related swelling (%)	30
Purulent saliva (%)	9
Pain (%)	12
Salty tasting saliva (%)	2
Number of swelling episodes in the past (%);	
Never	25
Once	23
Twice	23
Three or more	29
Antibiotic treatment needed (%)	17
Clinical examination	
Swelling (%)	49
Sensitivity in palpation (%)	22
Pain in palpation (%)	10
Gland secretion;	
Normal saliva (%)	50
Plaque (%)	2
Muddy (%)	5
Pus (%)	7
No secretion (%)	36
Palpation (%)	65
Gland involved	
Submandibular gland (%)	94
Parotid gland (%)	6
Right: Left (%)	48:52
Radiographic modality	
Ultrasound (%)	48
Panoramic X-ray (%)	7
Occlusal X-ray (%)	1
Computed tomography (%)	87
Sialography (%)	6
CT sialography (%)	9
Diagnostic endoscopy (%)	13
**Parameter**	**Value**
Sialolith characteristics (According to CT)	
Size(mm);	
Anterior-posterior (mean, SD)	6.6 ± 4.2
Superior-inferior (mean, SD)	7.4 ± 4.6
Lateral-lateral (mean, SD)	5.2 ± 2.8
Sialolith location (%);	
Gland	12
Hillus	44
Ductal	27
Papilla	17
Submandibular sialolith distance from fixed structures; (mean (mm), SD)	
Lingual border of the mandible	7.9 ± 3.7
Inferior border of the mandible	31 ± 7.5
Anterior border of the mandible	39 ± 18.2
Parotid sialolith distance from fixed structures; (mean (mm), SD)	
CEJ of the second maxillary molar	41.8 ± 6.9
External acoustic meatus	39.6 ± 6.8
**Operation**	
Duration of the operation (mean (sec), SD)	50 ± 15.6
Final results;	
Success VS. Failure (%)	87.4:12.6
**Post operation**	
Hospitalization (mean (days), SD)	2.57 ± 0.82
Complications (%)	5.5
**Follow-up**	
Duration; (mean (days), SD)	27.2, 19.6
Need for second attempt (%)	11

**Table 2:** (**a**): Results of linear regression analysis for all studied variables. (**b**): Results of linear regression analysis for the five variables accounting for 59% of the variance.

(a)									
	Unstandardized Coefficients		Standardized Coefficients	*t*	Significance	95.0% Confidence Interval for B		Collinearity Statistics	
	B	Standard Error	Beta			Lower Bound	Upper Bound	Tolerance	VIF
Age (years)	−0.003	0.016	−0.064	−0.197	0.847	−0.039	0.033	0.166	6.039
Gender (female)	−0.703	0.352	−0.476	−1.998	0.074	−1.486	0.081	0.309	3.24
Medications	0.109	0.308	0.083	0.353	0.731	−0.577	0.794	0.318	3.144
Past Symptoms (pain, salty saliva, plaques in saliva)	−0.162	0.403	−0.129	−0.401	0.697	−1.06	0.736	0.17	5.873
Number of past Swellings	0.001	0.209	0.001	0.003	0.998	−0.466	0.467	0.127	7.871
Past antibiotic treatment	1.234	0.658	0.502	1.875	0.09	−0.232	2.7	0.244	4.104
Gland swelling on admission	0.715	0.369	0.475	1.938	0.081	−0.107	1.536	0.291	3.434
Glan tender on palpation	0.03	0.669	0.017	0.045	0.965	−1.46	1.52	0.119	8.421
Saliva expression (0 = no saliva, 1 = clear, 2 = plaques, 3 = opaque, 4 = pus)	0.178	0.221	0.307	0.807	0.438	−0.314	0.671	0.121	8.277
Palpability of stone	0.712	0.681	0.427	1.045	0.32	−0.805	2.229	0.105	9.548
Side (left)	−0.696	0.486	−0.463	−1.433	0.182	−1.778	0.386	0.168	5.96
Type of imaging	0.136	0.387	0.092	0.352	0.732	−0.726	0.999	0.255	3.928
Number of imaging modalities	−0.048	0.263	−0.046	−0.183	0.859	−0.634	0.538	0.28	3.573
Sialolith volume	0	0	−0.103	−0.379	0.713	−0.001	0.001	0.235	4.254
Sialolith Location (1 = corpus of gland, 2 = hilum, 3 = central duct, 4 = papilla)	−0.067	0.19	−0.069	−0.352	0.732	−0.49	0.356	0.453	2.207
Surgery duration (minutes)	−0.013	0.015	−0.27	−0.888	0.395	−0.047	0.02	0.189	5.283
Success in stone removal	0.093	0.452	0.043	0.206	0.841	−0.914	1.1	0.403	2.484
(**b**)									
	**Unstandardized Coefficients**		**Standardized Coefficients**	***t***	**Significance**	**95.0% Confidence Interval for B**		**Collinearity Statistics**	
	**B**	**Standard Error**	**Beta**			**Lower Bound**	**Upper Bound**	**Tolerance**	**VIF**
Surgery duration (minutes)	0.017	0.007	0.342	2.501	0.019	0.003	0.032	0.843	1.186
Sialolith volume	0	0	−0.373	−2.763	0.01	−0.001	0	0.866	1.155
Past antibiotic treatment	1.01	0.369	0.401	2.735	0.011	0.251	1.769	0.732	1.366
Side (left)	−0.61	0.229	−0.403	−2.671	0.013	−1.08	−0.141	0.693	1.442
Age (years)	−0.015	0.007	−0.293	−2.194	0.037	−0.029	−0.001	0.886	1.129

Dependent variable: Number of hospitalization days.
